# Green spaces contribute to structural resilience of the gut microbiota in urban mammals

**DOI:** 10.1038/s41598-024-66209-4

**Published:** 2024-07-05

**Authors:** Rafał Łopucki, Ewa Sajnaga, Agnieszka Kalwasińska, Daniel Klich, Ignacy Kitowski, Dagmara Stępień-Pyśniak, Henrik Christensen

**Affiliations:** 1 Department of Biomedicine and Environmental Research, The John Paul II Catholic University of Lublin, Konstantynów 1J, 20-708 Lublin, Poland; 2 Department of Environmental Microbiology and Biotechnology, Nicolaus Copernicus University in Toruń, Lwowska 1, 87-100 Toruń, Poland; 3 Department of Animal Genetics and Conservation, Warsaw University of Life Sciences (SGGW), Ciszewskiego 8, 02-786 Warsaw, Poland; 4 University College of Applied Sciences in Chełm, Pocztowa 54, 22-100 Chełm, Poland; 5 Department of Veterinary Prevention and Avian Diseases, University of Life Sciences in Lublin, Głęboka 30, 20-612 Lublin, Poland; 6 Department of Veterinary and Animal Sciences, University of Copenhagen, Stigbøjlen 4, Frederiksberg C, Denmark

**Keywords:** Urbanization, Microbiota, 16S rRNA gene, Mammals, Microbiome, Wildlife, Ecology, Microbiology, Zoology, Environmental sciences

## Abstract

The gut microbiome of wild animals is subject to various environmental influences, including those associated with human-induced alterations to the environment. We investigated how the gut microbiota of a synurbic rodent species, the striped field mouse (*Apodemus agrarius*), change in cities of varying sizes, seeking the urban microbiota signature for this species. Fecal samples for analysis were collected from animals living in non-urbanized areas and green spaces of different-sized cities (Poland). Metagenomic 16S rRNA gene sequencing and further bioinformatics analyses were conducted. Significant differences in the composition of gut microbiomes among the studied populations were found. However, the observed changes were dependent on local habitat conditions, without strong evidence of a correlation with the size of the urbanized area. The results suggest that ecological detachment from a more natural, non-urban environment does not automatically lead to the development of an “urban microbiome” model in the studied rodent. The exposure to the natural environment in green spaces may serve as a catalyst for microbiome transformations, providing a previously underestimated contribution to the maintenance of native gut microbial communities in urban mammals.

## Introduction

Urbanization is a dynamic process of environmental transformation that significantly impacts biodiversity and ecosystem functioning^1^. This factor operates at multiple levels simultaneously and expose wildlife to combinations of stressors and stimuli unlike anything observed in nature^2^. Many species are unable to meet these challenges and exclude urbanized areas from their range of occurrence. However, there is a group of species, called urban adapters or synurbic species, which have adapted to urban life, successfully utilizing urban green spaces and various elements of urban infrastructure as habitats^3,4^. Animals inhabiting urban environments typically do not represent distinct species; more commonly, they form urban populations that have counterparts in traditional non-urban habitats. This prevalent habitat dualism provides unique opportunities for comparison of diverse behavioral, physiological, and genetic changes resulting from adaptation to urban environments^5–8^.

One of the significant consequences of the transition of animals to an urban lifestyle is a change in food preferences. The city offers a diverse array of food options, including naturally occurring sources; however, the facilitated access to human-produced food stands out as a predominant factor altering the feeding behavior and diet of urban animals^9–11^. A direct consequence of such dietary shifts may be the detrimental alterations in the microbial gut community^12^. These alterations may be further compounded by the direct or indirect impact of other urban factors, such as chemical, noise, and light pollution. Since the state of the gut microbiome is strongly associated with the physiology of the whole body^13–16^, its changes can have significant fitness and health repercussions for urban animals. In this context, alterations in the microbiome may constitute one of the pivotal factors determining the condition and adaptive success of animals in urban environments^12,17^.

To date, very few field studies have been conducted to demonstrate the actual transformations of the microbiome in urban mammal populations. For example, in their research conducted on Japanese macaques (*Macaca fuscata*), Lee et al.^18^ revealed that differences in the gut microbiome depend on the accessibility to anthropogenic food. It was also found that certain bacterial taxa can serve as indicators of the dependence of macaques on this source of food. Similarly, Sugden et al.^17^ demonstrated that increased consumption of carbohydrate-rich anthropogenic food by urban coyotes (*Canis latrans*) not only alters the gut microbiome (resulting in increased microbiome diversity and higher abundances of *Streptococcus* and *Enterococcus*) but also has negative effects on the overall health of the organisms. In another study, Anders et al.^19^ examined two rodent species with different dietary preferences and revealed that the dietary niche in urban areas is broader and may be linked to changes in the structure of gut microbial communities. However, microbiota changes strongly depended on the studied species: in one rodent, an increase in the abundance of potentially probiotic *Lactobacillus* was observed, while there was an increased abundance of potentially pathogenic *Helicobacter* in the other species. Also Stothart and Newman^12^ demonstrated a distinct impact of urbanization on the microbiome of urban populations of the eastern grey squirrel *Sciurus carolinensis*. They also identified a cluster of bacteria that characterize the microbiomes of squirrels inhabiting urbanized environments, probably associated with a low-fiber diet. However, they concluded that habitat heterogeneity within cities makes a representative ‘urban microbiome’ for the studied squirrel unlikely.

The presented examples indicate that field studies on microbiome alterations in urban mammals are limited and focus on a narrow range of species from various geographic regions. Simultaneously, the results obtained thus far are too diverse to construct reliable generalizations and infer how the plasticity of the microbial community composition and functions may enable the host to adapt to ecological changes induced by urbanization. There is also a lack of sufficient data to identify the most useful indicator groups of bacteria that could serve as ecological markers of the impact of urbanization on urban animals (but see^20^). Additionally, there are many other unexplored issues, such as the influence of the city size and habitat heterogeneity on the transformations of the microbiome in urban adapters.

The aim of this study was to examine urban-related alterations in the gut microbiome of the striped field mouse, *Apodemus agrarius*, a common synurbic rodent found in cities across Eastern Europe. We focused on exploring the potential correlation between the degree of microbiome alteration and the size of the urbanized area and identifying the taxonomic groups of bacteria that could serve as microbiota signature of urbanization in the studied rodent. We hypothesized that significant changes in the intestinal microbiota of the studied rodent will be observed already in populations living in small cities. We also assumed that these changes will become increasingly significant with the increasing degree of urbanization, i.e. the size of the city. Consequently, we expect that it will be possible to characterize the “urban microbiome” of the studied rodent and address the issue of its further adaptation to urban life.

The striped field mouse can serve as a valuable subject for studying urban-dependent transformations in the microbiome for several reasons. This species forms populations in both urban and non-urban areas, providing an opportunity for comparative studies on a large number of individuals. It occupies a broad dietary niche and can flexibly adapt the type of consumed food to habitat conditions, including utilization of anthropogenic food sources [described in details in^21^]. The small body size of this species allows it to inhabit even small urban green spaces, including green patches in the city center. Due to its limited mobility, this rodent is susceptible to the isolating effect of urban development and, consequently, dependent on local trophic conditions within the occupied urban green patches.

## Results

### Sequencing results

We conducted an analysis of the bacterial microbiota in 70 fecal samples categorized by both the sampling site (site groups: R, T, C, K, H, DS, and SK; N = 10 each) and the general place of origin (place groups: A—control, B—small city, C—medium-sized city, and D—large city; N = 20, except for group C for which N = 10) (compare Table 1; the term “group” refers to the “group of individuals” from which metabarcoding samples were collected). This analysis employed Illumina-based sequencing targeting the V3-V4 region of the 16S rRNA gene. The number of obtained high-quality DNA reads, approx. 430 bp long, ranged from 57,343 to 146,692, with a median of 109,086 for each sample (Table S1). The rarefaction curve analysis demonstrated that the sequencing data were sufficient to describe the bacterial diversity in the samples, as the curves obtained converged to a horizontal asymptote (Fig. S2). Out of the total 7,553,406 high-quality reads, ASVs were selected. The individual sample covered the number of ASVs in the range of 582–1447 (Table S1). There were 319 and 828 ASVs shared by all groups categorized by the sampling site and the place of origin, respectively (Fig. S3). A summary of data on assigning ASVs to bacterial taxonomy is presented in Table S2.

**Table 1:** Characteristic of the study sites.

General division of the study areas (place groups)	Description	Study sites acronyms and their latitude and longitude (site groups)	Habitat description
Place A Control areas	Rural area Typical habitat of occurrence of *Apodemus agrarius* in non-urbanized areas	Site R 51.291204°N, 22.640029°E	Narrow belts of semi-natural vegetation with grasses, herbs, shrubs, and single trees separating agricultural plots or desolate uncultivated area
		Site T 51.265085°N, 22.689259°E	
Place B Small city	Chełm City with population of about 60 thousand, area of 35 km^2^	Site K 51.138215°N, 23.484130°E	Urban park situated on the old cemetery
		Site C 51.141400°N, 23.476284°E	Green areas near railway line with trees and shrubs
Place C* Medium-sized city	Lublin City with population of about 350 thousand, area of 147 km^2^	Site H 51.218615°N, 22.565659°E	Green area with trees and shrubs within military and hospital area
Place D Big city	Warszawa City with population of about 1.8 million, area of 517 km^2^	Site SK 52.242020°N, 21.058915°E	Urban park with arranged vegetation and infrastructure (Skaryszewski Park)
		Site DS 52.168393°N, 21.033840°E	Urban naturalistic park with non-arranged vegetation (Służewiecka Valley)

*The place group C had half as many samples (N = 10) as the other place groups analyzed (N = 20).

### Bacterial community composition

At the phylum level, we identified a total of 43 taxa, with the number of taxa ranging from 16 to 31 in individual samples. Firmicutes and Bacteroidota emerged as the dominant phyla across all samples, constituting an average relative abundance (RA) of 69% and 19%, respectively. In contrast, Proteobacteria, Actinobacteriota, Desulfobacterota, and Patescibacteria represented 8%, 6%, 2%, and 1% of average RA, respectively. Other detected phyla exhibited less than 1% RA.

**Figure 1:**
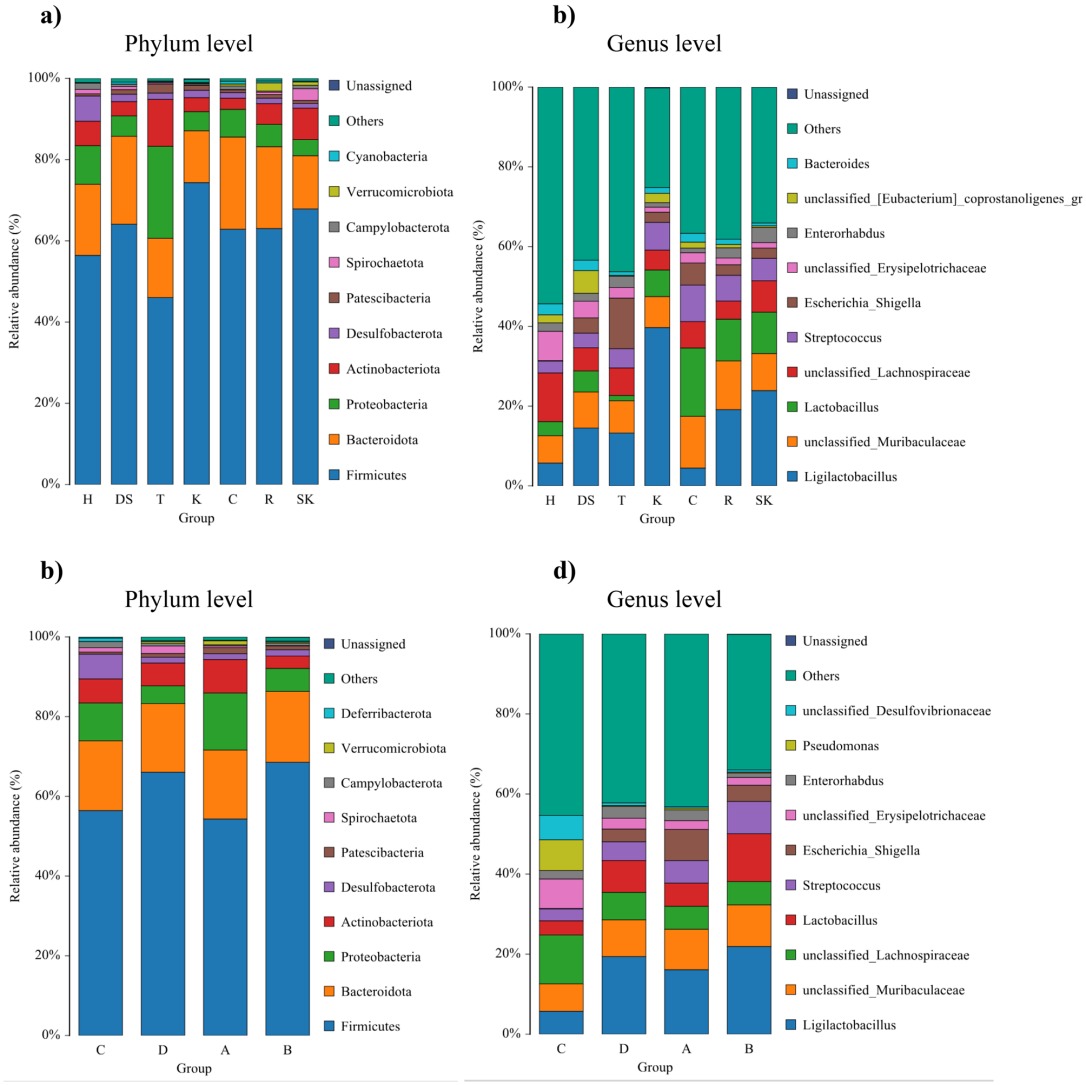
Relative abundance of bacterial communities at the phylum and genus level in groups categorized by the sampling site (**a**, **b**) and the place of origin (**c**, **d**). The abbreviations H, DS, T, K, C, R, and SK in charts a and b denote the acronyms of the sites where the rodents were captured and are described in detail in Table 1. The abbreviations A, B, C, and D in charts c and d denote rural areas, small city, medium-sized city, and large city, respectively.

At the genus level, we identified 1220 taxa in the entire dataset. The number of taxa at the genus level detected for individual samples ranged from 145 to 426, while only 34 bacterial taxa were present in all samples. However, these overlapping genera together accounted for 77% of average RA and were considered as a core microbiota. The prevalent genera were also the most abundant, including: *Ligilactobacillus* (16%), unclassified *Muribaculaceae* (10%), *Lactobacillus* (8%), unclassified *Lachnospiraceae* (7%), and *Streptococcus* (6%). Other genera accounted for ≤ 5% of average RA. The most abundant taxa at the phylum and genus level in each group categorized by both the place of origin and the sampling site are illustrated in Fig. 1.

### Variations in the microbiota diversity

**Figure 2:**
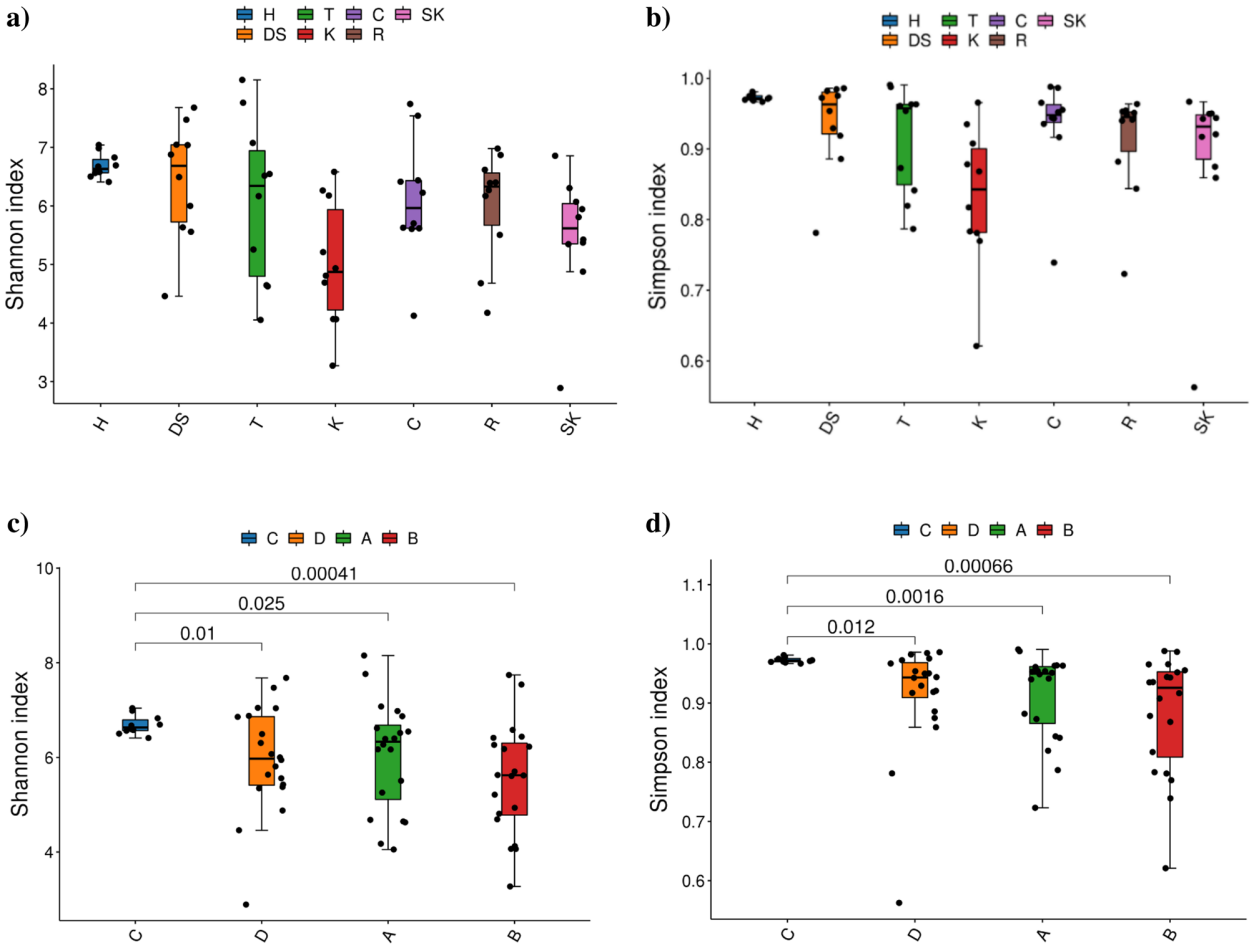
Shannon and Simpson indices of bacterial microbiota. The groups were categorized by the sampling site (**a**, **b**) and the place of origin (**c**, **d**). The P-value calculated by the T-test > 0.05 is not displayed by default. The abbreviations H, DS, T, K, C, R, and SK in charts a and b denote the acronyms of the sites where the rodents were captured and are described in detail in Table 1. The abbreviations A, B, C, and D in charts c and d denote: rural areas, small city, medium-sized city, and large city, respectively.

The alpha-diversity of the studied bacterial microbiota was assessed using diversity indices, i.e. Simpson and Shannon. Remarkably high values of α-diversity metrics were observed in all samples (Table S3). No statistically significant differences in richness and diversity were noted between groups of samples derived from the studied sampling sites (Fig. 2a,b). However, we found significant differences in Simpson and Shannon indices between the place C group (medium-sized city) and these from the other places of origin (Fig. 2c,d).

**Figure 3:**
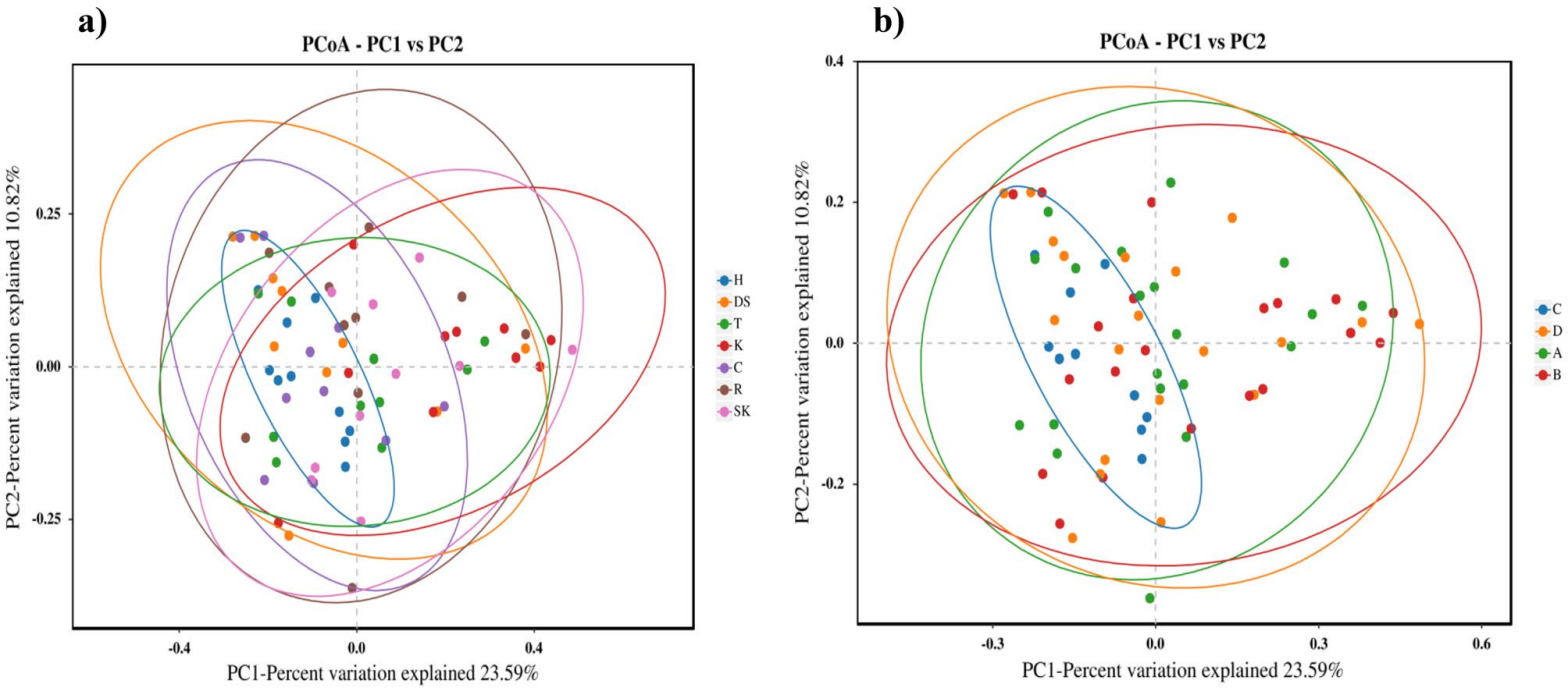
PCoA of bacterial microbiota based on weighted UniFrac distance showing the samples categorized by the sampling site (**a**) and the place of origin (**b**). The confidence ellipse defines the region containing 95% of all samples than can be drawn from the underlying Gaussian distribution. The abbreviations H, DS, T, K, C, R, and SK in chart a denote the acronyms of the sites where the rodents were captured and are described in detail in Table 1. The abbreviations A, B, C, and D in chart b denote: rural areas, small town, medium-sized city, and large city, respectively.

To assess the differences in the bacterial microbiota composition at the genus level, β-diversity was calculated using various statistical methods. PERMANOVA based on weighted UniFrac distance revealed significant differences between the experimental groups categorized by both the sampling site and the place of origin (P = 0.001) (Table S4). Additionally, we employed PCoA, which illustrated variations in sample dispersion (Fig. 3). Only samples derived from the medium-sized city (place C/site H) formed a distinct cluster, while the other samples exhibited greater dispersion in the ordination space.

### Differences in the microbiota composition between groups

**Figure 4:**
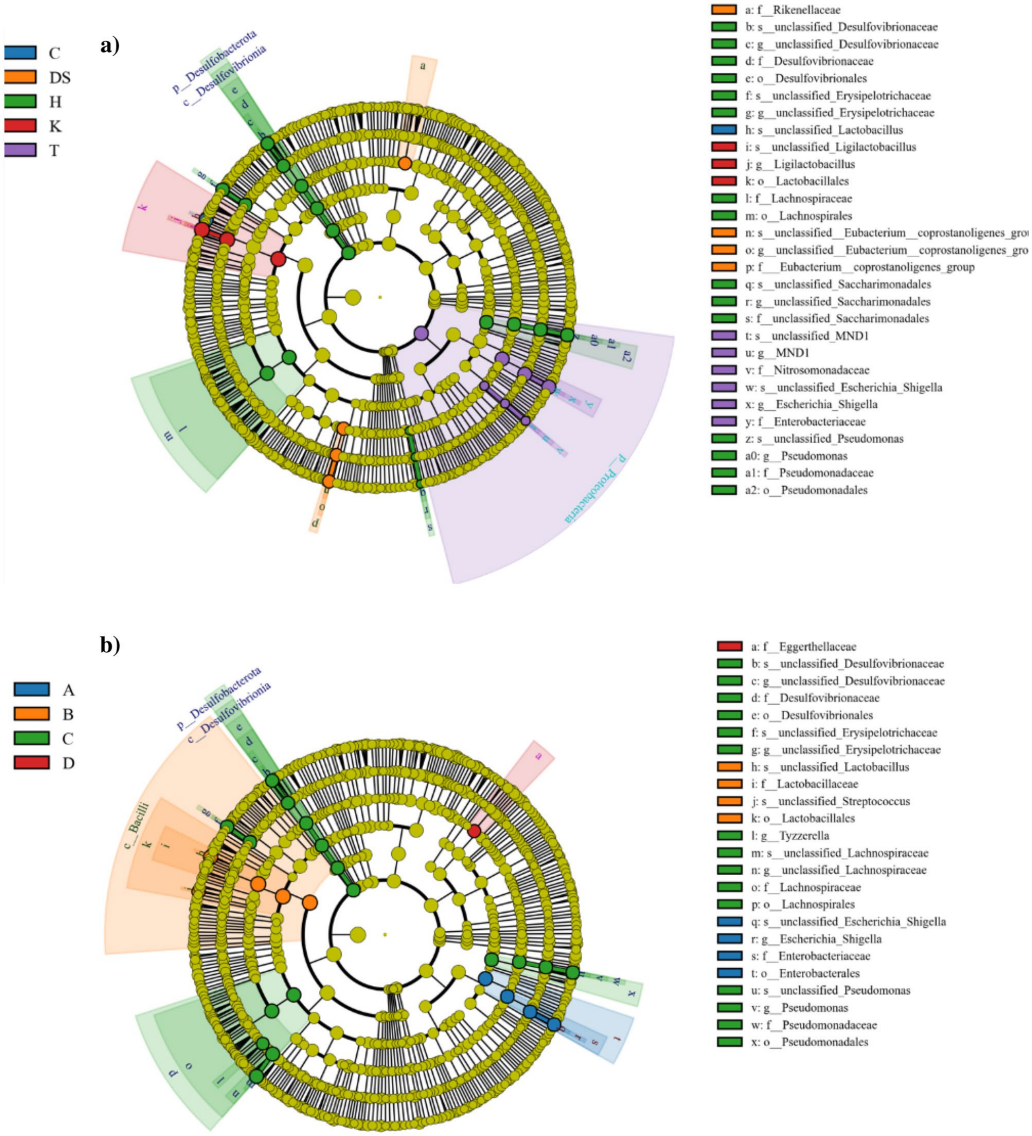
Cladograms obtained from the LEfSe analysis showing bacterial taxa with significantly different abundance values in groups categorized by the sampling site (**a**) and the place of origin (**b**). The circles represent the taxonomic level from the phylum to the species shown from the inside to the outside. The diameter of each circle is proportional to the taxon abundance. The yellow nodes represent taxa with no significant difference. Otherwise, the nodes are colored according to the group with the highest relative abundance. The abbreviations C, DS, H, K, and T in chart a denote the acronyms of the sites where the rodents were captured and are described in detail in Table 1. The abbreviations A, B, C, and D in chart b denote: rural areas, small city, medium-sized city, and large city, respectively.

The identification of taxa exhibiting significant changes in RA between the experimental groups was conducted through the LEfSe analysis. The results are presented in LEfSe cladograms showcasing bacterial taxa with significant RA differences among the groups (Fig. 4) and histograms displaying LDA scores indicating the effective size and ranking of each differentially abundant taxon (Fig. S4). When considering the groups categorized by the sampling site, the taxa abundance analysis revealed that the site H group (medium-sized city) exhibited the highest number of significant changes. The RA of the phylum Desulfobacterota and the families *Lachnospiraceae, Pseudomonadaceae*, and *Desulfovibrionaceae* was higher in the H site. At the genus level, the RA of *Pseudomonas*, unclassified *Desulfovibrionaceae*, unclassified *Erysipelotrichaceae*, and unclassified *Saccharimonadales* significantly increased in the H site. Conversely, in the site T group (control), we observed significantly elevated RA of representatives of the phylum Proteobacteria, families *Enterobacteriaceae* and *Nitrosomonadaceae*, and the genus level taxa *Escherichia/Shigella* and MND1. In contrast, no significant changes were detected in the other control site group (R). In the site K group (small city), a significant increase in RA was detected in the genus *Ligilactobacillu*s, while no significant variability was observed in the microbiota composition in the other site group from the small city (C). In the site DS group (big city), the analysis showed the greatest enrichment in the RA of the family *Rikenellaceae* and *Eubacterium coprostanoligenes* group. However, the other site group from the big city (SK) did no exhibit any significant changes in the RA of detected taxa compared to the other site groups (Fig. 4a, Fig. S4a).

According to the same LEfSe analysis, the comparison of the RA of groups categorized by the general place of origin confirmed that the place C group (medium-sized city) had the highest number of significant changes. This group exhibited greater abundance of the phylum Desulfobacterota and the families *Desulfovibrionaceae, Lachnospiraceae*, and *Pseudomonadaceae*. At the genus level, *Tyzella, Pseudomonas*, unclassified *Lachnospiraceae,* unclassified *Desulfovibrionaceae*, and unclassified *Erysipelotrichaceae* were significantly enriched in the place C group. In contrast, in the place A group (control), the RA of the family *Enterobacteriaceae* and the genus level taxon *Escherichia/Shigella* were significantly increased. Additionally, there was a significant increase in the RA of the families *Lactobacillaceae* in the place B group (small city) and *Eggerthellaceae* in the place D group (big city), compared to that of the other groups categorized by the place of origin (Fig. 4b, Fig. S4b).

### Functional analysis of the gut microbiota

**Figure 5:**
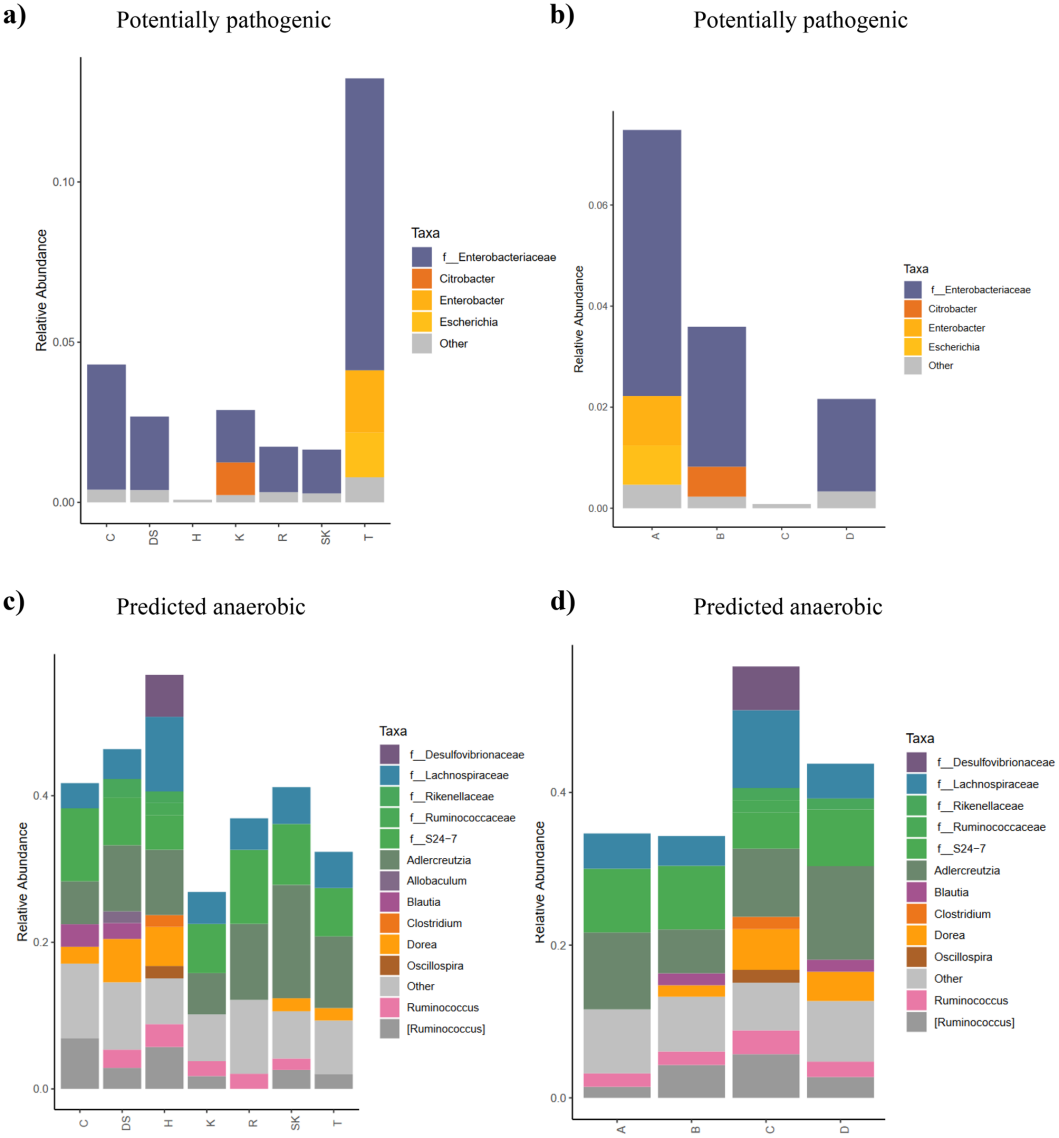
Histograms of BugBase predicted phenotypes of fecal microbiota in groups categorized by the sampling site (**a**, **c**) and the place of origin (**b**, **d**). Potentially pathogenic (**a**, **b**) and predicted anaerobic (**c**, **d**) taxa in each group are shown. The median estimates were compared across cohorts using the Kruskal–Wallis test and Pairwise Mann–Whitney-Wilcoxon tests for multiple comparisons. The abbreviations C, DS, H, K, R, SK, and T in charts a and c denote the acronyms of the sites where rodents were captured and are described in detail in Table 1. The abbreviations A, B, C, and D in charts b and d denote: rural areas, small town, medium-sized city, and large city, respectively.

We conducted phenotype predictions based on microbiome data using BugBase and compared the functional annotation between the experimental groups at the family level. The results revealed a significant decrease in the RA of potentially pathogenic bacteria in the site H group (medium-sized city) in comparison to the groups from the other sampling sites, except for the site K group (small city), where no difference was observed (FDR-corrected P value: H vs. DS < 0.001, H vs. R < 0.001, H vs. T = 0.002, H vs. C = 0.006, H vs. SK = 0.038) (Fig. 5a, Table S5a). Similarly, in groups categorized by the place of origin, the RA of potentially pathogenic bacteria was significantly reduced in the place C group (medium-sized city) (FDR-corrected P value: C vs. D < 0.001, C vs. A < 0.001, C vs. B = 0.005) (Fig. 5b, Table S5b). Most of the potentially pathogenic bacteria in all groups, except these from the medium-sized city (site H and place C groups), belonged to the family *Enterobacteriaceae*, with distinctive abundance of *Enterobacter* and *Escherichia* in the site T group (control) and *Citrobacter* in site K group (small city) (Fig. 5a, b). Due to a high drop in *Enterobacteriaece* RA, the groups from the medium-sized city also displayed a significant decrease in the RA of bacteria carrying mobile elements (data not shown).

Another phenotypic trait that significantly changed in the bacterial microbiota of the site H group (medium-sized city) was oxygen tolerance. Compared with the other groups categorized by the sampling site, the proportion of predicted anaerobic bacteria was significantly elevated in the site H group, except for the site SK and DS groups (big city) (FDR-corrected P value: H vs. K = 0.002, H vs. T = 0.007, H vs. R = 0.038, H vs. C = 0.04). The significantly higher RA of predicted anaerobic bacteria was also maintained in the site DS group, compared to the site K and T groups from the small city and the control area, respectively (FDR-corrected P value: DS vs. K = 0.007, DS vs. T = 0.038) (Fig. 5c, Table S5c). On the other hand, considering groups categorized by the place of origin, a significantly increased level of predicted anaerobic bacteria was again detected in the place C group (medium-sized city) (FDR-corrected P value: C vs. D < 0.001, C vs. A < 0.001, C vs. B = 0.05) (Fig. 5d, Table S5d). Most predicted anaerobic bacteria in the bacterial microbiota of the studied rodents originated from the families *Lachnospiraceae*, *Rikenellaceae,* and *Ruminococcaceae*; however, in the place C/site H groups (medium-sized city), which were the most enriched in predicted anaerobic bacteria, bacteria of the family *Desulfovibrionaceae* and the class Clostridia (*Clostridium, Oscillospira*) were additionally widespread (Fig. 5c,d). Taking into account the proportion of bacteria with other phenotypic features, such as those requiring oxygen, stress tolerance, and Gram staining, we did not find any significant differences among the groups.

### Correlations between urbanization of the environment and the gut microbiota composition

**Figure 6:**
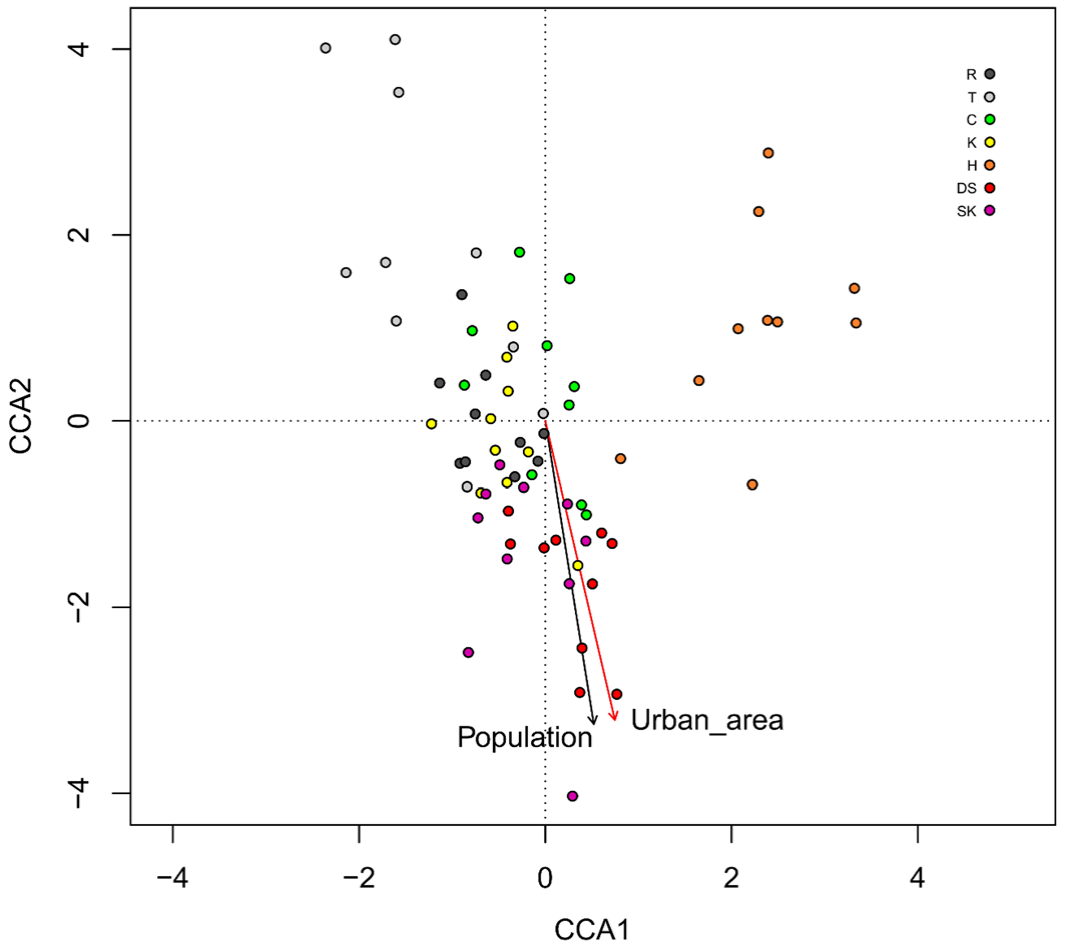
Relationship determined by means of canonical correspondence analysis (CCA) between anthropogenic pressure indicators (urban area and population) and bacterial variability at the ASV level. The most important and significant factor is marked in red. The abbreviations H, DS, T, K, C, R, and SK denote the acronyms of the sites where the rodents were captured and are described in detail in Table 1.

To analyze the potential association between the degree of urbanization and the bacterial microbiota composition at the genus level, we subsequently performed CCA analysis. The degree of urbanization in the sampling sites was described by two relatively objective variables: 1/the number of human inhabitants in a particular city (population) and 2/the administrative area of the city (urban area). It was shown that the tested CCA model was significant with P = 0.001. Additionally, the permutation test revealed that the latter anthropopressure factor (urban area) was significant (P = 0.001) while the former one was not significant (P = 0.105) in shaping the bacterial structure in the analyzed sites (Table S6, Fig. 6). To explore further the relationships between the size of the city and the frequencies of the top 100 abundant bacterial genera, we performed Spearman univariate correlation analysis. The results (Table S7) indicated a relatively low number of significant dependencies. The most evident relationships were as follows: the urban size was negatively correlated with the RA of *Pedobacter,* uncultured *Barnesiella*, and *Staphylococcus* (Rho = − 0.57, − 0.48, and − 0.47, respectively, P < 0.001) and positively correlated with the RA of the *Clostridium innocuum* group (Rho = 0.47, P < 0.001) (Table S7).

## Discussion

The gut microbiomes of wild animals are influenced by various environmental factors, and alterations in microbial communities enable the host to adapt more effectively to temporary or permanent changes in the environment^16,20,22,23^. In this context, the transformations of microbiomes in urban animals, as documented in the literature, are not only unsurprising but also anticipated. The outcomes of our research affirm that variations in microbiomes within urban habitats can also be observed in the striped field mouse—an urban adapter dwelling in green areas in the cities of Central and Eastern Europe. These alterations pertain to richness and diversity indices or shifts in the RA of specific bacterial taxon, among others. However, the question arises as to whether these changes are indeed the result of specific urban conditions or perhaps simply habitat differences, not necessarily related to urbanization. This is, therefore, a question about the mechanisms that determine microbiome transformations in urban areas and possibilities of reliable determination of the impact of urbanization on animal microbiomes in field experiments.

It is believed that the primary drivers behind microbiome transformation include, firstly, alterations in the diet (increased consumption of carbohydrates and protein in urban environments) and, secondly, the host's physiology and heightened stress levels resulting from a multitude of new evolutionary challenges posed to animals in urban areas^16^. It can, therefore, be inferred that the impact of these factors (and, consequently, the extent of microbiome transformation) will be more pronounced in larger than smaller cities. This inference stems from the observation that green spaces in larger cities undergo more significant transformation and isolation. Moreover, owing to the presence of a larger human population, the access to anthropogenic food sources may be more readily available. Consequently, it is reasonable to anticipate that, as cities increase in size and ecological separation from natural surroundings intensifies, changes in the microbiome composition accumulate, ultimately culminating in the formation of an “urban microbiome” within wild fauna. Regrettably, the correlation between the city size and the degree of microbiome transformation has not been extensively examined to date; even if some studies included comparisons between cities, these cities did not exhibit significant differences in size^12^.

Our research addresses the existing gap in knowledge and suggests that the city size does not exert a clear and directional influence on the gut bacterial microbiota of urban mammals. This holds true even when comparing cities with significant differences in size, ranging from 60,000 to 2 million inhabitants. The findings reveal that the differences in the composition of gut microbiota between the control rural groups of mice may be as significant as those between mice living in urban and rural areas, with the most substantial transformations observed in individuals not in the largest but in a medium-sized city. These outcomes underscore the pivotal role of local factors in shaping the microbiomes of the studied mammals. Such an influence of local factors is also clearly evident in more detailed analyses, for example, concerning the relative abundance of potential pathogens, which were most frequently found in the control areas rather than in urban areas. However, even the control areas showed significant differences in the obtained indices. This aligns with the findings reported by Stothart and Newman^12^, who investigated the microbiome of eastern gray squirrels (*Sciurus carolinensis*) and demonstrated that the variation explained by city-scale urbanization was comparable to the variation explained by land class heterogeneity within a single city.

The above considerations lead us also to the conclusion that the concept of the “urban microbiome” of the studied rodent may be challenging to delineate. Although we observe alterations in the gut microbiota composition within certain urban areas compared to non-urban areas and note significant differences among some cities, the trends of quantitative changes in bacterial genera related to the city size are modest and pertain to only a few bacterial taxon. The comparison of these results with the existing literature is challenging, as there is currently no clear consensus on which bacterial taxa can serve as indicators of microbiome “urbanity”. Various studies have identified different marker groups, such as *Lactobacillus* and *Helicobacter*^19^, *Akkermansiaceae* and *Lachnospiraceae*^12^, or *Enterobacterales*, *Deferribacterales*, and *Deferribacterales*^24^. Additionally, it is crucial to recognize that drawing analogies from research conducted on other mammalian species may be misleading, as Antwis et al.^25^ demonstrated significant differences in the composition of gut microbiota of closely related rodents, namely *A. agrarius*, *A. flavicollis*, and *A. sylvaticus*. Unfortunately, there is currently a lack of comprehensive metabarcoding data in the literature relating to the gut microbiota of rural or urban populations of the striped field mice (including^26^).

The most relevant comparative data illustrating the natural microbiome of field mice can be found in the study conducted by Antwis et al.^25^. Regrettably, the rodent population described in their work resides in a highly specific location—the Chernobyl Exclusion Zone (Ukraine) established after the nuclear power plant explosion. This area lacks current agricultural activities, and various habitats are undergoing different stages of ecological succession. Simultaneously, the resident rodents are exposed to radiation, making it unclear how this factor influences their gut microbiome. Consequently, drawing definitive conclusions through a comparison between the microbiome outlined in the Antwis et al.^25^ study and our research proves challenging; however, it is possible to note some similarities and differences describing the composition of the gut bacterial microbiota in field mice. For example, in the microbiota of Chernobyl mice, *Lachnospiraceae*, *Muribaculaceae*, *Ruminococceae*, *Lactobacillacae*, *Bacteroidaceae*, *Enterobacteriaceae*, *Rikenellaceae*, *Prevotellaceae*, *Erysipelotrichaceae*, *Desulfovibrionaceae*, and *Helicobacteraceae* were the most abundant families. On the other hand, in our research, we detected overlapping *Lactobacillaceae*, *Muribaculaceae*, *Lachnospiraceae*, *Streptococcaceae*, *Erysipelotrichaceae*, *Enterobacteriaceae*, *Desulfovibrionaceae*, and *Prevotellaceae* as well as distinctive *Eggerthellaceae*. This demonstrates a notable stability in the core groups of the gut microbiota among the studied species.

Taking all of the above into account, including the lack of significant differences between the rodents from the control areas and those from the largest city studied (e.g. R vs. SK), it can be inferred that the studied rodent species, even when inhabiting a large city, retains many features of the microbiome characteristic of populations from non-urbanized areas. This phenomenon is likely attributed to the fact that urban populations of striped field mice predominantly inhabit green areas. The dietary habits of animals residing in these green spaces may be more aligned with natural conditions, resulting in lower exposure to bacteria from humans and built environments. Consequently, the alterations in the microbiome composition of inhabitants in urban green areas may be less pronounced compared to synanthropic species, such as the house mouse or the brown rat. These synanthropic species utilize buildings and urban infrastructure for shelter and food and exhibit contact with specific human-associated bacteria^20,27^.

Currently, there are no comprehensive studies on the dietary habits of urban populations of the rodent species under investigation, and we did not address this aspect in our research. Some existing studies suggest that the diet of urban field mice may be more calorically dense^21^. Additionally, it has been proposed that this species experiences improved nutritional conditions in urban environments, as individuals captured during winter exhibited higher body weights compared to their rural counterparts^28^. Moreover, direct observations of urban mice revealed a lower inclination to defend food sources against fellow community members, potentially indicating easier access to food in urban settings^29^. Unfortunately, these findings only indirectly hint at the nutritional conditions in cities and cannot be used to quantitatively correlate the observed changes in the microbiome with the extent of urban diet transformation. Nevertheless, the strong affinity of urban populations of striped field mice to green spaces may suggest a continued connection between their diet and the natural food sources found in these areas. This observation may elucidate the phenomenon noted in our study, wherein urban and rural populations of the examined mice share approx. 77% similarity in the types of bacteria identified in their microbiomes. Consequently, these microbiome alterations can be considered relatively minor when compared to the potential changes described, for instance, in synanthropic species or following the transfer of wild animals to captivity^20,27^. However, even in captivity, where the diet undergoes significant changes and the exposure to diverse environmental bacteria is limited, mammals can retain a substantial portion of their native, species-specific microbiome^30^.

## Conclusions

Our research indicates that significant differences in the composition of the gut microbiota can be demonstrated when comparing both rural and urban striped field mouse populations. However, the observed changes depend on local habitat conditions, and no strong evidence was found to suggest that these changes are contingent upon the size of the urbanized area. The urban populations of the studied rodents, even in large cities, exhibited gut microbiomes similar to their counterparts living in non-urbanized areas. The observed similarity in the gut microbiota composition of urban populations, irrespective of the degree of urbanization, is likely attributable to the fact that these rodents in urban environments are closely associated with green areas and do not extensively utilize buildings and urban infrastructure, unlike synanthropic species. Consequently, their diet comprises a significant proportion of natural ingredients, while their exposure to human-derived bacteria is limited.

Our research supports the thesis that the microbiomes of urban mammal species that are strongly associated with green areas may exhibit different dependencies than those of species with a higher degree of synanthropization. This implies that ecological separation from more natural, non-urban surroundings does not automatically result in the development of an urban model for the wildlife microbiome. Urban green areas may play a crucial role as catalysts for such transformations. Considering green spaces as buffers for wild species to enter and inhabit cities may, therefore, offer an unappreciated contribution to the preservation of the natural gut microbiota communities of urban mammals.

## Methods

### Study area

We conducted the study in three cities of different size and in rural control areas, where occurrence of the striped field mouse were earlier experimentally confirmed. The biggest city was the capital of Poland—Warsaw (population of about 1.8 million; area of 517 km^2^), where two urban green areas were selected as study sites: Skaryszewski Park (site SK) and Służewiecka Valley (DS site). The medium-size city was Lublin (population of about 350 thousand; area of 147 km^2^), where one green area were selected (H site). The smallest city was Chełm (population of about 60 thousand, area of 35 km^2^), where two green areas were selected (K and C sites). The control sites were located within the agriculture fields near Lublin in two separated locations (R and T sites). Detailed characteristics of the study areas and their location were presented in Table 1 and in Figure S1.

### Collection of fecal samples from rodents

As a research samples, we used fresh feces collected from live-captured rodents. This method is suitable for investigating the microbiota of the lower gastrointestinal tract in rodents^31^. To obtain fecal samples small mammals were captured in live traps with oats as bait. In each of the study areas the traps were set along a transect and spaced about 10 m from one another. Traps were checked up to 6 h after setting. Captured animals were described in terms of species, sex, and body mass, and released at the site of capture. A fresh faecal samples defecated by the rodents during handling procedures was collected to a sterile Eppendorf. The sample was described, cooled in a portable transport refrigerator, transported to the laboratory and stored at − 80 °C until further analysis. Trapping was conducted in winter 2022/2023. The study material collected during field trapping comprised 70 samples, with 10 samples obtained from each study site.

### DNA extraction

After thawing, the fecal samples were vigorously mixed and 0.1 g each sample was transferred to sterile Eppendorf tube. Bacterial DNA were extracted using a PureLink Microbiome DNA Purification Kit (Invitrogen, Carlsband, USA) according stool samples protocol provided by manufacturer. The concentration and purity of the isolated DNA were subsequently check with Qubit 2 fluorometer (Invitrogen, Carlsband, USA) using a Qubit dsDNA HS Assay kit (Thermo Fisher Scientific, Waltham, USA) and PicoDrop spectrofotometer (Picodrop, UK), respectively.

### High throughput 16S rRNA gene sequencing

Metagenomic next generation sequencing (NGS) and bioinformatics analysis were performed by BMKGene (Biomarker Technologies, Beijing, China). High-quality of provided DNA samples was confirmed during sample quality control. The hypervariable region V3-V4 of the bacterial 16S rRNA gene were amplified with primers 338F: 5'- ACTCCTACGGGAGGCAGCA-3' and 806R: 5'- GGACTACHVGGGTWTCTAAT-3'. The primers were tailed with Illumina index sequences allowing for deep sequencing. The PCR was performed using high-fidelity KOD FX Neo DNA polymerase (TOYOBO Biotech, Shanghai, China) according to the manufacturer’s instruction. PCR products were purified with Omega DNA purification kit (Omega Inc., Norcross, GA, USA) and quantified using Qsep-400 (BiOptic, Inc., New Taipei City, Taiwan). Sequencing was performed on a Novaseq 6000 platform (Illumina, San Diego, USA) in a pair end-mode. The bioinformatics analysis was carried out with the aid of the BMKCloud (http://www.biocloud.net/). Raw data was primarily filtered by Trimmomatic ver. 0.33. Identification and removal of primer sequences was processed by Cutadapt ver. 1.9.1. Pair-end reads obtained from previous steps were assembled by USEARCH ver. 10 and followed by chimera removal using UCHIME ver. 8.1. Denoising and generating amplicon sequence variants (ASV) was carried out using DADA2 method^32^ in QIIME2. Taxonomy annotation of ASVs was performed using the SILVA database ver. 138 based on the Naive Bayes classifier in QIIME2. Sequencing data were deposited in NCBI under accession number PRJNA1060930.

### Data availability

Sequencing data were deposited in NCBI under accession number PRJNA1060930 (https://www.ncbi.nlm.nih.gov/bioproject/PRJNA1060930/).

### Exploratory data analysis

Rarefaction curves, calculations of alpha diversity metric, Venn diagrams, principal coordinate analysis (PCoA based on UniFrac distance) and permutational multivariate analysis of variance (PERMANOVA) were performed with QIIME2 (https://qiime2.org/). Differences in the α diversity metrics between experimental groups were evaluated with t-test and visualized by using Metastats software (http://metastats.cbcb.umd.edu/). Linear discriminant analysis (LDA) coupled with effect size (LEfSe) (http://huttenhower.sph.harvard.edu/lefse/) was employed to test the significant taxonomic difference among groups^33^. A logarithmic LDA score of 4.0 was set as the threshold for discriminative features. BugBase algorithm was used for phenotype type predictions (https://bugbase.cs.umn.edu/), such as pathogenic potential, oxygen requiring, Gram staining, and contents of mobile elements^34^. The pairwise Mann–Whitney-Wilcoxon test was used to obtain the differentially abundant bacteria with an FDR-corrected P value less than 0.05, while non-parametric factorial Kruskal–Wallis (KW) sum-rank test aimed at detecting significant differences in taxa abundance among experimental groups. Vegan package in R (ver. 4.0.3) was implemented for canonical correspondence analysis (CCA) to calculate association between gut bacteria composition and anthropopressure factors. Permutation test for CCA model was performed in the same R package. The single effects of urban area on faecal microbiota were evaluated in detail on the basis of Spearman correlations with Hmisc package in R (ver. 4.4.0).

### Ethical statement

According to the Polish regulations for the realized sampling no ethical permission was required. In accordance with Polish regulations, the capture of small mammals of unprotected species in live traps for research/monitoring purposes, including biometric measurements and determination of their systematic affiliation, does not require the consent of the bioethics committee (Article 1.2.4 of the Act of 15 January 2015 on the protection of animals used for scientific or educational purposes, Dz. U. 2015 poz. 266). During the study, the rodents were not subjected to any procedures other than temporary handling to determine species, body weight and sex. Animals were not relocated.

### Supplementary Information

Supplementary Information.
