# Supplementary material for: Green spaces contribute to structural resilience of the gut microbiota in urban mammals
Source: Sci Rep. 2024 Jul 5;14:15508. doi: 10.1038/s41598-024-66209-4 (PMC11226671; doi:10.1038/s41598-024-66209-4)
Supplement: Supplementary file 1 — Supplementary Information. [file 41598_2024_66209_MOESM1_ESM.docx]

**Table S1.** Summary on quality control of reads in each sample and resultant ASVs

| **Sample ID** | **Raw reads** | **Clean reads** | **Denoised reads** | **Merged reads** | **Non-chimeric reads** | | **Feature sequences** | | **ASVs** | |
| --- | --- | --- | --- | --- | --- | --- | --- | --- | --- | --- |
| C11 | 160060 | 145194 | 144752 | 138584 | 110490 | 109931 | | 757 | |  |
| C14 | 160023 | 144878 | 144531 | 139508 | 104210 | 103735 | | 675 | |  |
| C16 | 159867 | 145759 | 145247 | 139387 | 104406 | 103815 | | 717 | |  |
| C20 | 160178 | 145465 | 144935 | 140302 | 96045 | 95574 | | 624 | |  |
| C21 | 159753 | 145299 | 144850 | 140556 | 103675 | 103191 | | 683 | |  |
| C3 | 159838 | 145185 | 144883 | 141598 | 116864 | 116371 | | 762 | |  |
| C4 | 160132 | 144604 | 143941 | 133743 | 112727 | 111769 | | 1227 | |  |
| C5 | 159823 | 144929 | 144266 | 133450 | 114254 | 113445 | | 1122 | |  |
| C6 | 160182 | 145648 | 145076 | 137344 | 106563 | 105738 | | 1007 | |  |
| C7 | 159809 | 145065 | 144589 | 138453 | 121002 | 120387 | | 974 | |  |
| DS1 | 167435 | 153133 | 152117 | 130765 | 63654 | 61951 | | 1161 | |  |
| DS13 | 159846 | 143033 | 142496 | 138195 | 125898 | 124867 | | 987 | |  |
| DS15 | 160096 | 144687 | 143965 | 137256 | 118036 | 117009 | | 1187 | |  |
| DS17 | 159877 | 145608 | 145094 | 138925 | 117552 | 116889 | | 800 | |  |
| DS19 | 159721 | 144291 | 143354 | 128549 | 103227 | 101944 | | 1447 | |  |
| DS2 | 160024 | 146339 | 145683 | 135845 | 98634 | 97720 | | 873 | |  |
| DS21 | 159990 | 145541 | 144901 | 135205 | 104274 | 103374 | | 1087 | |  |
| DS22 | 160313 | 146211 | 145666 | 135709 | 106178 | 105426 | | 1053 | |  |
| DS7 | 160251 | 145028 | 144569 | 135230 | 107874 | 107142 | | 825 | |  |
| DS8 | 160225 | 145550 | 144851 | 131028 | 98781 | 97797 | | 1181 | |  |
| H12 | 159912 | 145093 | 144660 | 138148 | 110766 | 110070 | | 820 | |  |
| H13 | 159828 | 145611 | 144912 | 138683 | 108745 | 107999 | | 886 | |  |
| H14 | 160475 | 146817 | 146334 | 140944 | 112147 | 111653 | | 729 | |  |
| H18 | 160050 | 144272 | 143917 | 137110 | 111169 | 110593 | | 818 | |  |
| H19 | 159922 | 144772 | 144107 | 134200 | 109180 | 108419 | | 994 | |  |
| H20 | 160331 | 146375 | 145715 | 136172 | 104683 | 103982 | | 927 | |  |
| H21 | 160109 | 146832 | 146238 | 139872 | 101511 | 100911 | | 782 | |  |
| H3 | 160032 | 147065 | 146309 | 131693 | 89349 | 88292 | | 971 | |  |
| H8 | 159659 | 145064 | 144599 | 139429 | 110417 | 109753 | | 746 | |  |
| H9 | 160208 | 146268 | 145880 | 139478 | 112251 | 111757 | | 672 | |  |
| K11 | 160023 | 145092 | 144630 | 136898 | 86278 | 85572 | | 712 | |  |
| K15 | 159618 | 144301 | 143531 | 133248 | 100721 | 99010 | | 1374 | |  |
| K16 | 159824 | 143727 | 143486 | 139306 | 116776 | 116306 | | 700 | |  |
| K17 | 160180 | 145817 | 145331 | 140985 | 103407 | 102798 | | 737 | |  |
| K19 | 160088 | 144780 | 144251 | 140097 | 111792 | 111203 | | 671 | |  |
| K20 | 170914 | 153714 | 153396 | 150549 | 147338 | 146692 | | 838 | |  |
| K21 | 161833 | 148189 | 147426 | 135993 | 58431 | 57343 | | 807 | |  |
| K26 | 160158 | 146149 | 145571 | 140228 | 115261 | 114750 | | 819 | |  |
| K4 | 160081 | 143050 | 142646 | 138842 | 118513 | 117889 | | 695 | |  |
| K6 | 159823 | 143750 | 143297 | 138066 | 112563 | 111900 | | 725 | |  |
| R12 | 159574 | 145793 | 145242 | 139221 | 112358 | 111614 | | 858 | |  |
| R14 | 159897 | 145320 | 144737 | 137460 | 101428 | 100619 | | 825 | |  |
| *continuation* |  |  |  |  |  |  | |  | |  |
| **Sample ID** | **Raw reads** | **Clean reads** | **Denoised reads** | **Merged reads** | **Non-chimeric Reads** | **Feature sequences** | | **ASVs** | |  |
| R23 | 159884 | 143971 | 143277 | 136775 | 117729 | 116655 | | 1106 | |  |
| R25 | 160146 | 145493 | 144852 | 139044 | 104515 | 103829 | | 925 | |  |
| R3 | 160073 | 143894 | 143475 | 139152 | 95668 | 94984 | | 591 | |  |
| R5 | 159912 | 144931 | 144270 | 139314 | 106371 | 105600 | | 855 | |  |
| R6 | 159491 | 144759 | 144107 | 136995 | 98568 | 97592 | | 941 | |  |
| R7 | 159928 | 145169 | 144592 | 137577 | 106513 | 105725 | | 900 | |  |
| SK1 | 160164 | 147124 | 146783 | 140282 | 105625 | 105192 | | 582 | |  |
| SK10 | 160093 | 145307 | 144702 | 136466 | 118771 | 118039 | | 1038 | |  |
| SK13 | 159685 | 145940 | 145260 | 135739 | 117204 | 116452 | | 1179 | |  |
| SK14 | 160003 | 144451 | 144044 | 140139 | 123805 | 123276 | | 809 | |  |
| SK15 | 160276 | 145553 | 144863 | 136570 | 102252 | 101498 | | 813 | |  |
| SK2 | 159925 | 144599 | 144074 | 140084 | 119174 | 118207 | | 979 | |  |
| SK3 | 160242 | 145416 | 144858 | 141307 | 117053 | 116451 | | 1080 | |  |
| SK5 | 160027 | 145553 | 145162 | 139710 | 106469 | 105873 | | 767 | |  |
| SK6 | 159760 | 145477 | 145059 | 138552 | 92215 | 91666 | | 618 | |  |
| SK8 | 159879 | 143685 | 143388 | 139705 | 128765 | 128147 | | 843 | |  |
| T11 | 159890 | 144046 | 143558 | 138311 | 120341 | 119266 | | 997 | |  |
| T12 | 159818 | 143934 | 143533 | 139093 | 119747 | 118745 | | 845 | |  |
| T18 | 160300 | 146454 | 145638 | 135254 | 99345 | 98432 | | 1110 | |  |
| T19 | 160159 | 145243 | 144744 | 138424 | 105054 | 104311 | | 800 | |  |
| T20 | 159901 | 145549 | 145023 | 138269 | 113608 | 112903 | | 1003 | |  |
| T24 | 160043 | 144527 | 143910 | 138982 | 120722 | 119859 | | 980 | |  |
| T25 | 160184 | 145236 | 144775 | 139411 | 111262 | 110353 | | 879 | |  |
| T27 | 159908 | 146026 | 145144 | 134092 | 114573 | 113606 | | 1262 | |  |
| T3 | 160068 | 143706 | 143108 | 137581 | 119861 | 118682 | | 1047 | |  |
| T4 | 160002 | 146526 | 145891 | 136882 | 104061 | 103289 | | 937 | |  |
|  |  |  |  |  | **In total:** | **7553406** | | **34469** | |  |


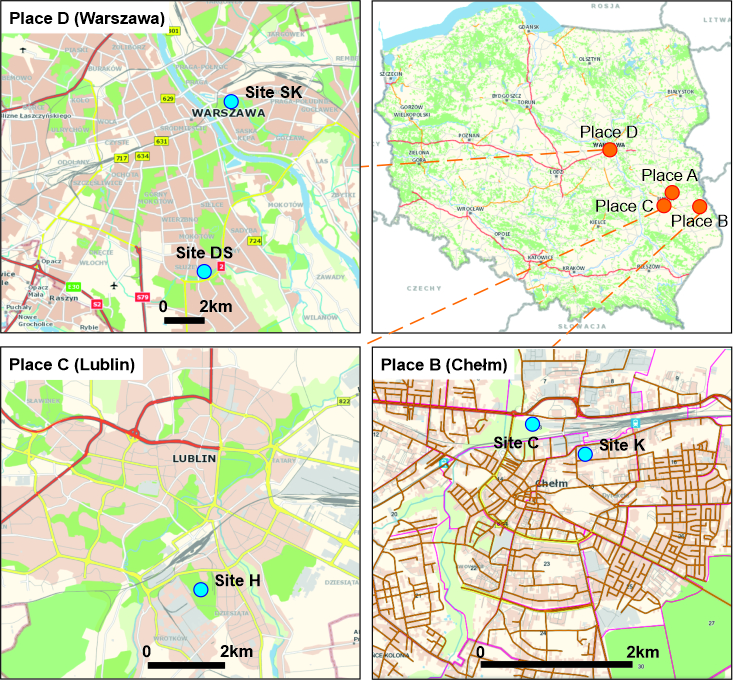


**Fig. S1** General location of the control (A) and urban (B, C, D) study sites in Poland, with detailed locations of the urban study sites in green areas within a small city (Chełm), a medium-sized city (Lublin), and a large city (Warsaw). Additional descriptions of the study areas are provided in Table 1. The figure was generated in CorelDRAW Standard 2020 (https://www.corel.com), and the map background comes from National Geoportal service (https://mapy.geoportal.gov.pl/imap/Imgp_2.html).

**a)**


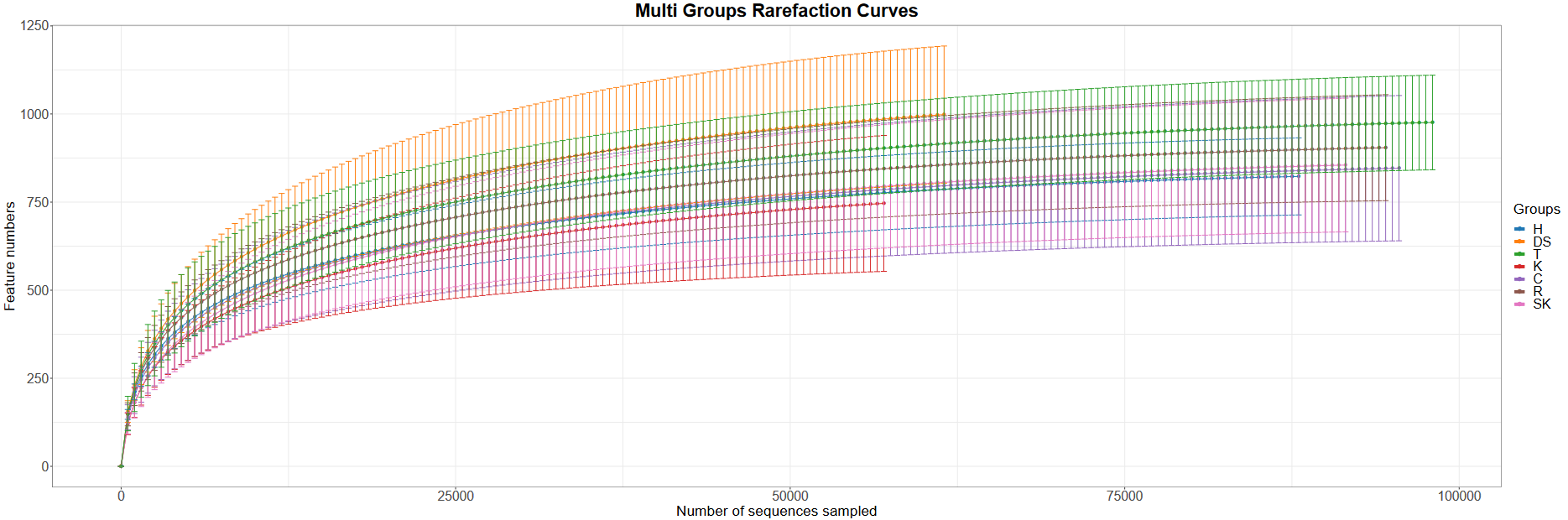


**b)**


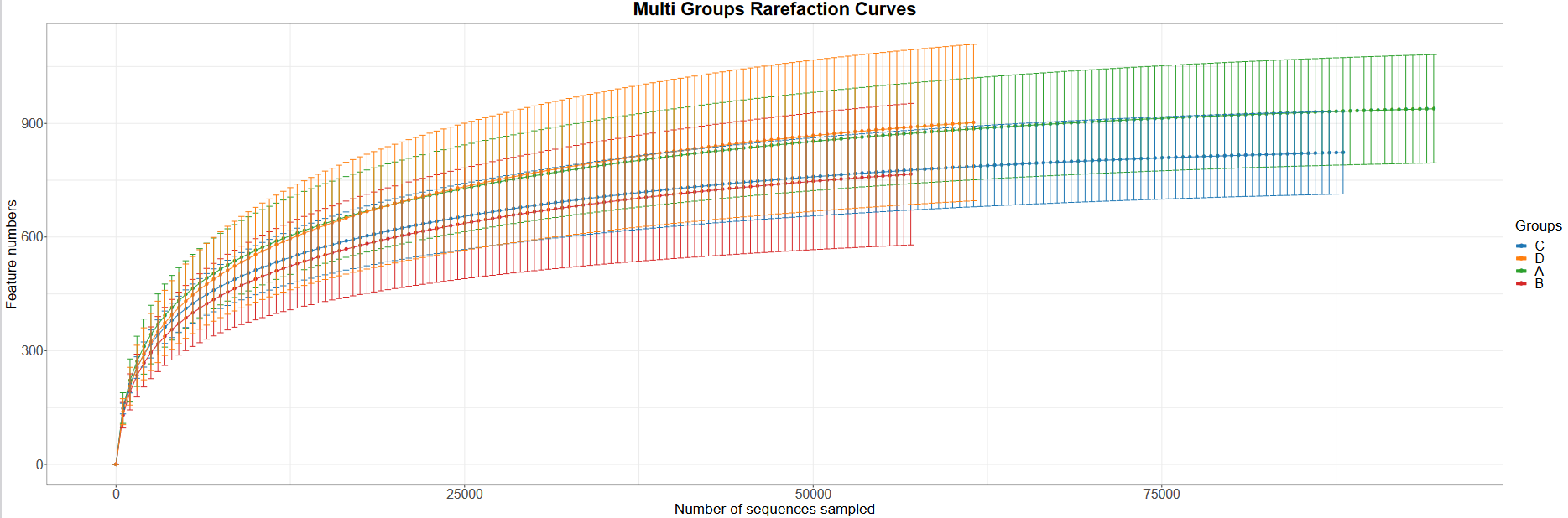


**Fig. S2** Rarefaction curves in groups categorized by the sampling site (a) and the place of origin (b).

| The abbreviations H, DS, T, K, C, R, and SK denote the acronyms of the sites where rodents were captured and were described in detail in Table 1. The abbreviations A, B, C and D denote, respectively: rural areas, small town, medium-sized city, and large city. |  |  |  |  |  |  |  |  |  |  |  |  |  |  |  |  |  |  |  |  |  |  |  |
| --- | --- | --- | --- | --- | --- | --- | --- | --- | --- | --- | --- | --- | --- | --- | --- | --- | --- | --- | --- | --- | --- | --- | --- |
|  | | | | | | | | | | | | | | | | | | | | | | | |

**a)**


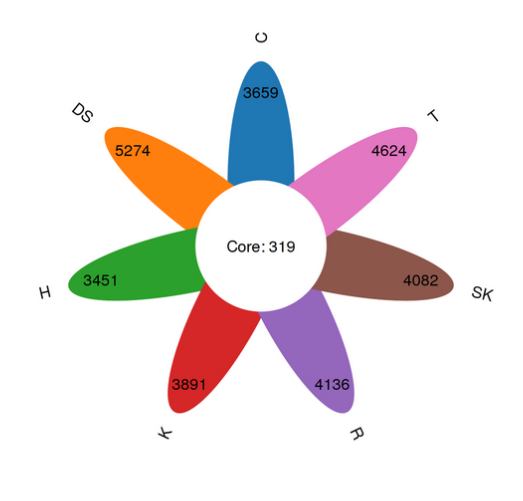


**b)**


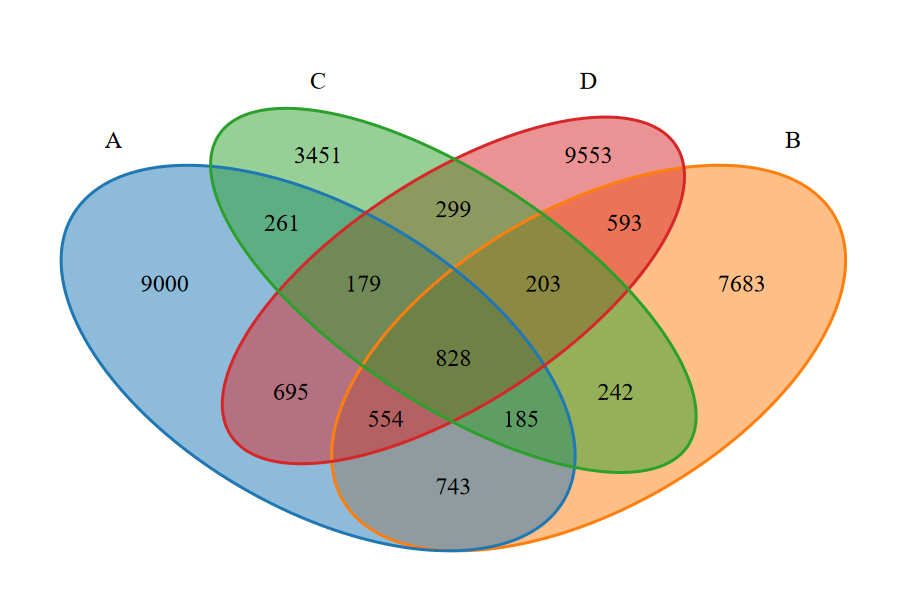


**Fig. S3.** Venn diagrams showing unique and overlapping ASVs among groups categorized by the sampling site (a) and the place of origin (b).

| The abbreviations H, DS, T, K, C, R, and SK denote the acronyms of the sites where rodents were captured and were described in detail in Table 1. The abbreviations A, B, C and D denote, respectively: rural areas, small town, medium-sized city, and large city. |  |
| --- | --- |
|  |  |
|  | |

**Table S2.** Statistics of reads in each taxonomic level for each sample

| **Sample** | **Kingdom** | **Phylum** | **Class** | **Order** | **Family** | **Genus** | **Species** |
| --- | --- | --- | --- | --- | --- | --- | --- |
| C11 | 109931 | 109913 | 109904 | 109829 | 108848 | 84764 | 16595 |
| C14 | 103735 | 103703 | 103677 | 103552 | 102057 | 82114 | 12292 |
| C16 | 103815 | 103791 | 103767 | 103640 | 101651 | 75163 | 11970 |
| C20 | 95574 | 95548 | 95542 | 94922 | 93956 | 70037 | 7758 |
| C21 | 103191 | 103173 | 103170 | 99707 | 98067 | 72207 | 4228 |
| C3 | 116371 | 115870 | 115854 | 115571 | 113241 | 103845 | 19105 |
| C4 | 111769 | 110607 | 110568 | 109922 | 104181 | 51066 | 13725 |
| C5 | 113445 | 112977 | 112966 | 112439 | 104974 | 55930 | 13230 |
| C6 | 105738 | 105346 | 105314 | 105091 | 101506 | 72286 | 39788 |
| C7 | 120387 | 119730 | 119712 | 119352 | 115233 | 69043 | 20019 |
| DS1 | 61951 | 61846 | 61809 | 54102 | 49621 | 27106 | 4117 |
| DS13 | 124867 | 124785 | 124734 | 124167 | 122158 | 103307 | 3789 |
| DS15 | 117009 | 116954 | 116899 | 115697 | 107250 | 65640 | 32947 |
| DS17 | 116889 | 116180 | 116171 | 115850 | 112866 | 82345 | 25398 |
| DS19 | 101944 | 100928 | 100845 | 100255 | 88545 | 56317 | 11138 |
| DS2 | 97720 | 97695 | 97677 | 96525 | 91045 | 65978 | 18359 |
| DS21 | 103374 | 102736 | 102694 | 101828 | 98864 | 73277 | 11808 |
| DS22 | 105426 | 104483 | 104439 | 104244 | 98731 | 55232 | 29952 |
| DS7 | 107142 | 106442 | 106439 | 106174 | 104219 | 70386 | 8984 |
| DS8 | 97797 | 96692 | 96665 | 96462 | 92299 | 44533 | 9449 |
| H12 | 110070 | 110025 | 109980 | 109757 | 106411 | 70839 | 9803 |
| H13 | 107999 | 107894 | 107826 | 107609 | 105277 | 77368 | 8845 |
| H14 | 111653 | 111600 | 111576 | 109569 | 108170 | 84329 | 9083 |
| H18 | 110593 | 110540 | 110523 | 110144 | 108312 | 61249 | 7811 |
| H19 | 108419 | 108376 | 108324 | 107881 | 105321 | 52062 | 4112 |
| H20 | 103982 | 103948 | 103930 | 103259 | 100035 | 46769 | 5813 |
| H21 | 100911 | 100876 | 100868 | 96898 | 91688 | 56480 | 6135 |
| H3 | 88292 | 88213 | 88196 | 86790 | 82685 | 39256 | 4774 |
| H8 | 109753 | 109709 | 109704 | 108127 | 107001 | 57032 | 11411 |
| H9 | 111757 | 111704 | 111672 | 109514 | 107854 | 63005 | 8597 |
| K11 | 85572 | 85531 | 85529 | 84362 | 83792 | 61434 | 13945 |
| K15 | 99010 | 98800 | 98721 | 98544 | 96477 | 76992 | 2716 |
| K16 | 116306 | 116275 | 116258 | 115907 | 115120 | 101754 | 3750 |
| K17 | 102798 | 102711 | 102699 | 97407 | 94820 | 76267 | 4024 |
| K19 | 111203 | 111189 | 111180 | 109352 | 108393 | 86875 | 59483 |
| K20 | 146692 | 146093 | 146078 | 145679 | 141312 | 113789 | 14636 |
| K21 | 57343 | 57229 | 57196 | 55782 | 53285 | 35512 | 3777 |
| K26 | 114750 | 114692 | 114677 | 107684 | 102451 | 56726 | 4029 |
| K4 | 117889 | 117862 | 117833 | 116805 | 116200 | 104168 | 4095 |
| K6 | 111900 | 111842 | 111813 | 111352 | 110188 | 99855 | 3077 |
| R12 | 111614 | 111541 | 111529 | 95066 | 87987 | 71142 | 10195 |
| R14 | 100619 | 100576 | 100556 | 99407 | 89237 | 55309 | 28585 |
| *continuation* |  |  |  |  |  |  |  |
| **Sample** | **Kingdom** | **Phylum** | **Class** | **Order** | **Family** | **Genus** | **Species** |
| R18 | 103804 | 103757 | 103750 | 103079 | 94809 | 65116 | 8913 |
| R22 | 123770 | 123521 | 123462 | 121225 | 119302 | 107703 | 9450 |
| R23 | 116655 | 116557 | 116513 | 114969 | 111266 | 86381 | 8082 |
| R25 | 103829 | 103775 | 103771 | 99286 | 92026 | 56792 | 29410 |
| R3 | 94984 | 94942 | 94932 | 88264 | 87213 | 78537 | 40163 |
| R5 | 105600 | 105553 | 105530 | 104747 | 99619 | 63266 | 3818 |
| R6 | 97592 | 97566 | 97541 | 87532 | 83673 | 54629 | 14637 |
| R7 | 105725 | 105685 | 105671 | 102632 | 97038 | 75379 | 34508 |
| SK1 | 105192 | 105176 | 105174 | 90182 | 88767 | 55059 | 27160 |
| SK10 | 118039 | 117434 | 117370 | 117165 | 106829 | 74139 | 2422 |
| SK13 | 116452 | 115490 | 115410 | 115199 | 111947 | 80021 | 12168 |
| SK14 | 123276 | 122622 | 122618 | 122349 | 121309 | 95738 | 39994 |
| SK15 | 101498 | 100748 | 100722 | 99452 | 97720 | 70275 | 16467 |
| SK2 | 118207 | 118092 | 118068 | 108562 | 106439 | 85459 | 14163 |
| SK3 | 116451 | 116359 | 116324 | 114705 | 105158 | 77136 | 28680 |
| SK5 | 105873 | 105810 | 105766 | 97018 | 95052 | 77103 | 43594 |
| SK6 | 91666 | 91644 | 91629 | 85125 | 83184 | 61544 | 26049 |
| SK8 | 128147 | 128112 | 128085 | 126138 | 124965 | 115010 | 1258 |
| T11 | 119266 | 119165 | 119120 | 118816 | 115425 | 91432 | 8449 |
| T12 | 118745 | 118679 | 118643 | 118238 | 117548 | 96711 | 1398 |
| T18 | 98432 | 98348 | 98338 | 96951 | 92883 | 44897 | 10179 |
| T19 | 104311 | 104282 | 104267 | 102832 | 98682 | 67230 | 19337 |
| T20 | 112903 | 112828 | 112820 | 111849 | 108996 | 66224 | 31067 |
| T24 | 119859 | 119776 | 119763 | 118890 | 113210 | 91712 | 23429 |
| T25 | 110353 | 110267 | 110221 | 109794 | 108261 | 94714 | 3798 |
| T27 | 113606 | 112462 | 112432 | 108900 | 102286 | 63564 | 15077 |
| T3 | 118682 | 118545 | 118502 | 117739 | 115695 | 90896 | 5439 |
| T4 | 103289 | 102963 | 102931 | 102482 | 98421 | 76432 | 10483 |

**Table S3.** Alpha diversity indices in each sample

| **Sample ID** | **Simpson** | **Shannon** |
| --- | --- | --- |
| C11 | 0.9440 | 5.6070 |
| C14 | 0.9431 | 5.7026 |
| C16 | 0.9553 | 6.2259 |
| C20 | 0.9354 | 5.6273 |
| C21 | 0.9166 | 5.6179 |
| C3 | 0.7391 | 4.1254 |
| C4 | 0.9879 | 7.7422 |
| C5 | 0.9864 | 7.5396 |
| C6 | 0.9519 | 6.4380 |
| C7 | 0.9653 | 6.4138 |
| DS1 | 0.9752 | 7.0395 |
| DS13 | 0.7812 | 4.4599 |
| DS15 | 0.9187 | 6.0005 |
| DS17 | 0.9292 | 5.6353 |
| DS19 | 0.9859 | 7.6800 |
| DS2 | 0.9723 | 6.8771 |
| DS21 | 0.8858 | 5.5591 |
| DS22 | 0.9537 | 6.4930 |
| DS7 | 0.9820 | 7.0465 |
| DS8 | 0.9844 | 7.4715 |
| H12 | 0.9684 | 6.5787 |
| H13 | 0.9667 | 6.4106 |
| H14 | 0.9695 | 6.5019 |
| H18 | 0.9723 | 6.6927 |
| H19 | 0.9700 | 6.6723 |
| H20 | 0.9710 | 6.8281 |
| H21 | 0.9755 | 7.0417 |
| H3 | 0.9792 | 6.9867 |
| H8 | 0.9747 | 6.5626 |
| H9 | 0.9810 | 6.5922 |
| K11 | 0.8681 | 4.9356 |
| K15 | 0.7832 | 4.8104 |
| K16 | 0.7696 | 4.0648 |
| K17 | 0.8782 | 5.2119 |
| K19 | 0.8172 | 4.6917 |
| K20 | 0.9077 | 6.1784 |
| K21 | 0.9655 | 6.5815 |
| K26 | 0.9349 | 6.2641 |
| K4 | 0.7810 | 4.0678 |
| K6 | 0.6210 | 3.2718 |
| R12 | 0.9544 | 6.2701 |
| R14  *continuation* | 0.9528 | 6.1708 |
| **Sample ID** | **Simpson** | **Shannon** |
| R22 | 0.7231 | 4.1751 |
| R23 | 0.8437 | 5.5048 |
| R25 | 0.9510 | 6.9793 |
| R3 | 0.8819 | 4.6813 |
| R5 | 0.9400 | 6.6181 |
| R6 | 0.9486 | 6.3917 |
| R7 | 0.9413 | 6.4004 |
| SK1 | 0.9502 | 5.8097 |
| SK10 | 0.8748 | 5.9456 |
| SK13 | 0.9668 | 6.8566 |
| SK14 | 0.8591 | 4.8775 |
| SK15 | 0.9497 | 6.0704 |
| SK2 | 0.9171 | 5.3468 |
| SK3 | 0.9426 | 6.3056 |
| SK5 | 0.9207 | 5.3743 |
| SK6 | 0.9438 | 5.4259 |
| SK8 | 0.5625 | 2.8897 |
| T11 | 0.8729 | 5.2559 |
| T12 | 0.8195 | 4.0541 |
| T18 | 0.9906 | 8.1531 |
| T19 | 0.9539 | 6.1686 |
| T20 | 0.9609 | 7.0757 |
| T24 | 0.9631 | 6.5166 |
| T25 | 0.8413 | 4.6267 |
| T27 | 0.9876 | 7.7630 |
| T3 | 0.7868 | 4.6464 |
| T4 | 0.9631 | 6.5454 |

**Table S4**. PERMANOVA based on weighted UniFrac distance for bacterial communities at the genus level between groups categorized by the place of origin and the sampling site

|  | | | | |  |  |
| --- | --- | --- | --- | --- | --- | --- |
|  |  |  |  |  |  |  |
|  | Df | Sums of sqs | Mean sqs | F. Model | R2 | P value |
| Place | 3 | 0.994 | 0.331 | 2.49 | 0.101 | 0.001 |
| Site | 6 | 2.002 | 0.334 | 2.70 | 0.204 | 0.001 |

**a)**


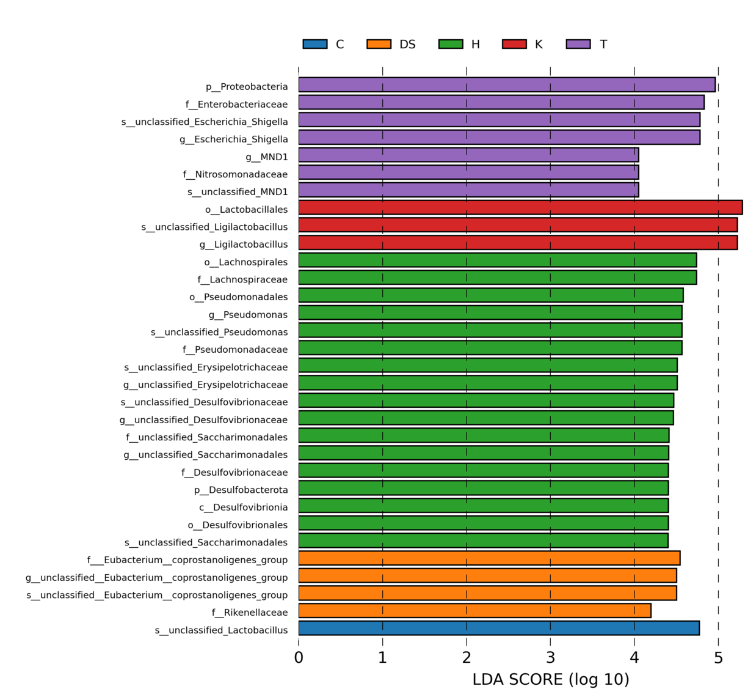


**b)**


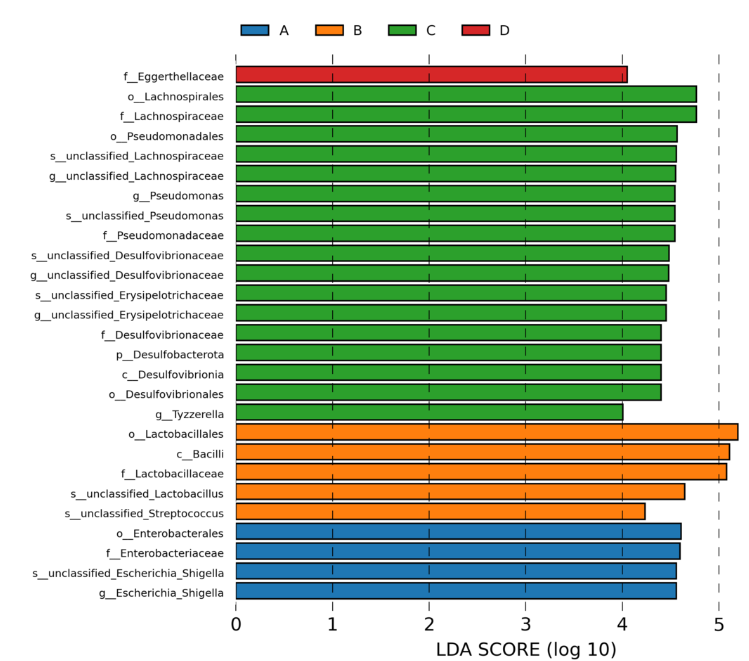


**Fig. S4**. Histogram of the linear discriminant analysis (LDA) scores which indicates the effective size and ranking of each differentially abundant taxon. The comparison was performed between groups categorized by the sampling site (a) and the place of origin (b).

The abbreviations H, DS, T, K, and C, denote the acronyms of the sites where rodents were captured and were described in detail in Table 1. The abbreviations A, B, C and D denote, respectively: rural areas, small town, medium-sized city, and large city.

**Table S5.** FDR-corrected pairwise p-values and group p-values being results of Pairwise Mann-Whitney-Wilcoxon and Kruskal-Wallis tests, respectively, showing the difference of the relative abundance of potentially pathogenic (a, b) and predicted anaerobic (c, d) bacteria between groups categorized by the sampling site (a, c) and the place of origin (b, d). The relative abundance of potentially pathogenic and predicted anaerobic bacteria was determined using the BagBase algorithm (<https://bugbase.cs.umn.edu/>) as described in the section *Materials and methods*.

1. **potentially pathogenic bacteria, sampling site**

| H_vs_DS | H_vs_T | H_vs_K | H_vs_C | H_vs_R | H_vs_SK | DS_vs_T | DS_vs_K | DS_vs_C | DS_vs_R |  |  | |  | |  |
| --- | --- | --- | --- | --- | --- | --- | --- | --- | --- | --- | --- | --- | --- | --- | --- |
| 0.000 | 0.002 | 0.138 | 0.006 | 0.000 | 0.038 | 0.367 | 0.367 | 0.776 | 0.776 |  |  | |  | |  |
|  |  |  |  |  |  |  |  |  |  |  | |  | |  | |
| DS_vs_SK | T_vs_K | T_vs_C | T_vs_R | T_vs_SK | K_vs_C | K_vs_R | K_vs_SK | C_vs_R | C_vs_SK | R_vs_SK | |  | |  | |
| 0.436 | 0.124 | 0.367 | 0.367 | 0.138 | 0.367 | 0.367 | 0.776 | 0.853 | 0.367 | 0.367 | |  | |  | |

Group p-value: 0.000658

1. **potentially pathogenic bacteria, place of origin**

| C_vs_D | C_vs_A | C_vs_B | D_vs_A | D_vs_B | A_vs_B |  |
| --- | --- | --- | --- | --- | --- | --- |
| 0.000 | 0.000 | 0.005 | 0.121 | 0.989 | 0.130 |  |

Group p-value: 0.000154

1. **predicted anaerobic bacteria, sampling site**

| H_vs_DS | H_vs_T | H_vs_K | H_vs_C | H_vs_R | H_vs_SK | DS_vs_T | DS_vs_K | DS_vs_C | DS_vs_R | DS_vs_SK |
| --- | --- | --- | --- | --- | --- | --- | --- | --- | --- | --- |
| 0.187 | 0.007 | 0.002 | 0.040 | 0.038 | 0.130 | 0.038 | 0.007 | 0.305 | 0.147 | 0.389 |
|  |  |  |  |  |  |  |  |  |  |  |
| T_vs_K | T_vs_C | T_vs_R | T_vs_SK | K_vs_C | K_vs_R | K_vs_SK | C_vs_R | C_vs_SK | R_vs_SK |  |
| 0.817 | 0.285 | 0.325 | 0.231 | 0.231 | 0.147 | 0.231 | 0.912 | 0.912 | 0.509 |  |

Group p-value: 0.000733

1. **predicted anaerobic bacteria, place of origin**

| C_vs_D | C_vs_A | C_vs_B | D_vs_A | D_vs_B | A_vs_B |
| --- | --- | --- | --- | --- | --- |
| 0.041 | 0.001 | 0.001 | 0.029 | 0.048 | 0.799 |

Group p-value: 0.000412

**Table S6.** Results of the permutation test for CCA analysis

|  | **Df** | **Chi Square** | **F** | **Pr(>F)** |
| --- | --- | --- | --- | --- |
| **Population** | 1 | 0.0710 | 1.2903 | 0.105 |
| **Urban area** | 1 | 0.1743 | 3.1685 | 0.001*** |

Signif. codes: 0 ‘***’ 0.001 ‘**’ 0.01 ‘*’ 0.05 ‘.’ 0.1 ‘ ’ 1

**Table S7**. Significant associations between the relative abundance of the 100 top bacterial genera in fecal microbiota and the size of the urbanized area

| Taxon | Speraman's rho | p-value |
| --- | --- | --- |
| Pedobacter | -0.57 | 0.000 |
| Uncultured Barnesiella sp. | -0.48 | 0.000 |
| Staphylococcus | -0.47 | 0.000 |
| Carnobacterium | -0.42 | 0.000 |
| Turicibacter | -0.40 | 0.001 |
| Unclassified Rickettsiales | -0.36 | 0.002 |
| Lysobacter | -0.35 | 0.003 |
| Brachybacterium | -0.32 | 0.008 |
| Unclassified Bacteroidia | -0.31 | 0.009 |
| Akkermansia | -0.29 | 0.014 |
| Unclassified Rs E47 termite group | -0.29 | 0.016 |
| Escherichia Shigella | -0.28 | 0.020 |
| Streptococcus | -0.27 | 0.021 |
| Unclassified Paracaedibacteraceae | -0.27 | 0.026 |
| Unclassified Enterobacteriaceae | -0.25 | 0.034 |
| Unclassified Rhodospirillales | -0.25 | 0.035 |
| Lactococcus | 0.24 | 0.045 |
| Candidatus Stoquefichus | 0.24 | 0.042 |
| Mucispirillum | 0.25 | 0.034 |
| Monoglobus | 0.26 | 0.033 |
| Helicobacter | 0.26 | 0.027 |
| Treponema | 0.27 | 0.022 |
| Prevotellaceae UCG_003 | 0.28 | 0.020 |
| Unclassified Erysipelatoclostridiaceae | 0.33 | 0.005 |
| Quinella | 0.34 | 0.004 |
| Unclassified Desulfovibrionaceae | 0.36 | 0.003 |
| Unclassified [Eubacterium] coprostanoligenes group | 0.38 | 0.001 |
| Adlercreutzia | 0.38 | 0.001 |
| Bacillus | 0.39 | 0.001 |
| Unclassified Paludibacteraceae | 0.40 | 0.001 |
| Unclassified Prevotellaceae | 0.45 | 0.000 |
| [Clostridium] innocuum group | 0.47 | 0.000 |
